# Pharmacological and Interventional Prevention and Treatment of Microvascular Obstruction Following Primary PCI in STEMI

**DOI:** 10.3390/jcdd12110440

**Published:** 2025-11-07

**Authors:** Giovanni Occhipinti, Michele Strosio, Riccardo Rinaldi, Andrea Ruberti, Salvatore Brugaletta

**Affiliations:** 1 Cardiology Department, Cardiovascular Clinic Institute, Hospital Clínic de Barcelona, University of Barcelona, 08193 Barcelona, Spain; 2 Cardiology Unit, Department of Cardiac-Thoracic-Vascular Sciences and Public Health, University of Padova, Via Nicolò Giustiniani 2, 35128 Padova, Italy

**Keywords:** STEMI, microvascular obstruction, MVO, coronary no-Reflow, PCI

## Abstract

Microvascular obstruction (MVO) accounts for up to 50% of patients diagnosed with ST-elevation myocardial infarction (STEMI) undergoing primary percutaneous coronary intervention (PCI). The pathogenesis is multifactorial and includes myocardial ischemia, distal embolization, and ischemia–reperfusion injury, in a context of individual susceptibility. Its occurrence has been related to adverse outcomes. Despite an extensive body of research, no single pharmacological or interventional strategy has proven effectiveness. The inconclusive nature of the evidence can be attributed to lack of standardization among studies in terms of drugs used and their dosage, the variability in study designs, and the fact that available studies performed a decade ago feature reperfusion strategies and drugs used that differ significantly from the current standard of care. In this context, our review aims to discuss the pharmacological and interventional approaches to MVO.

## 1. Introduction

Microvascular obstruction (MVO) is a highly dynamic process characterized by failure to adequately reperfuse the microvascular bed despite successful epicardial coronary artery reperfusion [1]. First described in 1948 by Harman and colleagues in albino male rabbits, its impact on adverse clinical outcomes was highlighted more than two decades ago [2,3]. MVO after epicardial vessel reperfusion can be explained by a joint action of at least four factors: myocardial ischemia, spontaneous or iatrogenic distal embolization, reperfusion-related injury, and individual susceptibility. These factors contribute to (i) intravascular obstruction (e.g., endothelial cell swelling, platelet–neutrophil plugging, fibrin-thrombus generation, etc.), and (ii) extravascular compression (e.g., myocardial edema, cardiomyocyte swelling/hyper-contracture, intramyocardial hemorrhage, pericyte contracture, etc.). Due to the absence of a standardized diagnostic approach, data on its incidence are not homogeneous; nevertheless, it may occur in approximately 40–50% of patients undergoing percutaneous coronary intervention (PCI), with a higher incidence among patients with ST-elevation myocardial infarction (STEMI) [4,5]. MVO is a strong contributor to poor clinical outcomes [6,7].

Over the last three decades, extensive efforts have been made to identify pharmacological and interventional approaches targeting the mechanisms of MVO at various stages of primary PCI (pPCI), with a major focus on targeting intravascular causes of MVO; however, no satisfactory therapy has been found to prevent or reverse this phenomenon and to consistently improve clinical outcomes [8,9]. The inconclusive nature of the evidence can be attributed to the lack of standardization among studies in terms of drugs used and their dosage, the substantial variability in study designs, and the relatively old evidence from studies conducted over a decade ago when access to care and reperfusion strategies differed significantly from current standards of care.

In this context, this review provides a comprehensive overview on the pharmacological and interventional treatments, highlighting the existing clinical gaps in the field. It also provides a view on ongoing clinical trials and studies.

## 2. Mechanisms of Microvascular Obstruction

The mechanism behind MVO is multifactorial and involves the interplay of multiple factors [8]. Among those, myocardial ischemia, spontaneous or iatrogenic distal embolization, and ischemia–reperfusion injury are the major determinants [10]. Additionally, individual susceptibility—presence of conditions predisposing to microvascular dysfunction (i.e., advanced age, male sex, diabetes mellitus, etc.)—plays an important role [11].

**Figure 1:**
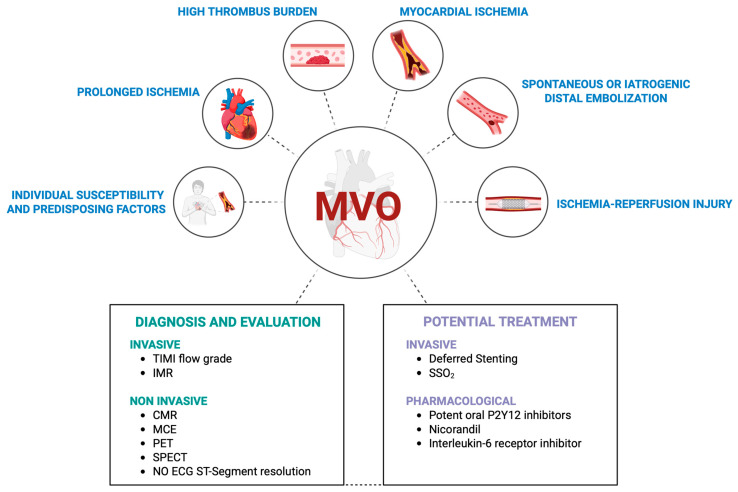
Comprehensive summary of microvascular obstruction (MVO): causes, diagnosis, and promising treatments for MVO from randomized trials. Abbreviations: TIMI: Thrombolysis in Myocardial Infarction; IMR: Index of Myocardial Resistance; CMR: cardiac magnetic resonance; MCE: Myocardial Contrast Echocardiography; PET: Positron Emission Tomography; SPECT: Single-Photon Emission Computed Tomography; SSO_2_: Supersaturated Oxygen. (e.g., Science Suite Inc. dba BioRender, created in BioRender, Strosio, M. (2025). https://BioRender.com/yrdruj6 (accessed on 1 November 2025)).

Each of these mechanisms, together with factors such as prolonged ischemia and high thrombus burden, contributes to the complex interplay of events leading to impaired microvascular perfusion and subsequent myocardial injury [12]. The discussion on the mechanisms of MVO is out of the scope of this review and has been extensively described [8] (Figure 1).

## 3. Diagnosis of Microvascular Obstruction

**Figure 2:**
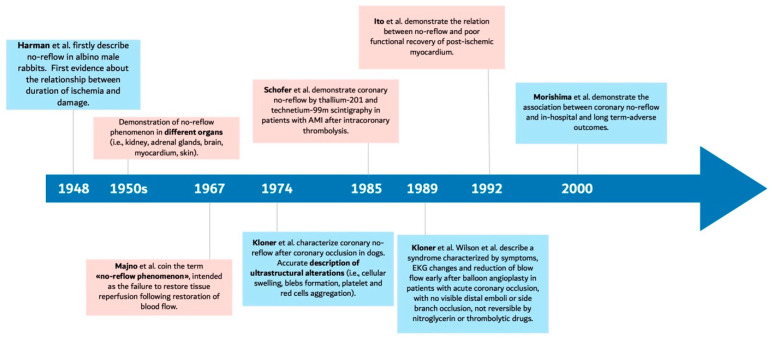
The story of microvascular obstruction over the years.

The underlying pathological mechanism of MVO can develop gradually and progress over hours following the restoration of coronary blood flow and may persist for weeks, as firstly described by Schofer and colleagues, who demonstrated the persistence of MVO up to 4 weeks after intracoronary thrombolysis [13] (Figure 2). Progression and duration of MVO depend on the severity and extent of myocardial ischemia, as well as effectiveness of therapeutic measures implemented to prevent or mitigate injury. The diagnostic effectiveness depends on its extent, severity, and the timing of evaluation. Coronary angiography through angiographical metrics such as Thrombolysis in Myocardial Infarction (TIMI) flow grade, corrected TIMI frame count (CTFC), myocardial blush grade (MBG), and TIMI myocardial perfusion grade has been largely used as a tool to diagnose MVO. Although angiographic-based tools can quickly assess MVO, their reliability for detection of MVO is scarce [14,15]. This is because MVO can also occur in the absence of normal post-procedural angiography metrics (i.e., TIMI flow grade or myocardial blush grade) [16,17]. In addition, the early timing of assessment of these indices, made at the time of coronary angiography, does not adequately assess the high dynamism of the phenomenon, which develops during the days after the event [18]. As a result, it has been reported that by using TIMI grading criteria or their combination with TIMI myocardial perfusion grade (a TIMI flow grade ≤2 or a TIMI flow grade of 3 with a TIMI myocardial perfusion grade of 0–1), MVO can be diagnosed in up to 29% and 32% of patients, respectively. Other techniques with a higher sensitivity include cardiac magnetic resonance (CMR), as well as nuclear imaging techniques such as Single-Photon Emission Computed Tomography (SPECT) and Positron Emission Tomography (PET). CMR imaging-based detection of MVO is defined based on a lack of contrast uptake during the first pass of contrast agent (imaging obtained within one minute after injection), lack of early gadolinium enhancement (imaging obtained within 2–3 min after injection) or lack of late gadolinium enhancement (imaging 10–15 min after contrast injection) [14,19,20]. Using CMR imaging, MVO was diagnosed in up 57% of patients with STEMI within 7 days after pPCI by late gadolinium enhancement imaging and diagnosed in up to 95% of patients with STEMI, as well as a restored TIMI flow grade of 3 during first-pass contrast-enhanced imaging [6]. Moreover, CMR not only identifies MVO, but also provides prognostic information by detecting intramyocardial hemorrhage and by estimating infarct size. Myocardial scintigraphy offered the first evidence on the existence of MVO in humans and holds historical importance as a diagnostic tool [13]. Among nuclear imaging techniques, the diagnostic performance of PET is higher; nevertheless, this technique has limited use in daily clinical practice due to technical challenges, limited expertise to interpret results, high costs, and radiation exposure. Other techniques include Myocardial Contrast Echocardiography (MCE), employing gas-filled microbubbles as tracers to visualize microcirculation [21], and invasive coronary physiology measurements, such as coronary flow velocity patterns and index of microvascular resistance. Nevertheless, these techniques have limited use for the diagnosis of CNR in current clinical practice [22,23]. Finally, ST-segment resolution upon electrocardiogram (ECG) has also been used as a diagnostic tool, but its sensitivity is lower compared to other imaging techniques [24].

## 4. Pharmacological Prevention and Treatment of Microvascular Obstruction

Various pharmacological strategies have been proposed to prevent and treat MVO in patients with STEMI [8]. Among these, antithrombotic agents [oral and parenteral antiplatelet agents, parenteral anticoagulants, intracoronary (IC) antithrombotic agents] and vasodilators (adenosine, calcium channel blockers, nitroprusside, nicorandil) have been extensively studied in randomized controlled trials (RCTs), evaluating their efficacy against placebo or in head-to-head comparisons [9]. However, no effective therapy has been identified so far to prevent or treat MVO. Table 1 shows tested pharmacological molecules.

### 4.1. Adenosine

Adenosine, a purine nucleoside generated by purine synthesis or adenosine triphosphate breakdown, exerts a potent vasodilatory effect on the coronary arterioles and microcirculation via A2A receptor binding on endothelial cells [25]. By targeting neutrophil aggregation, it also reduces neutrophilic arterial plugging, a central mechanism of ischemia–reperfusion injury, and promotes vascular repair following vascular injury [26]. Adenosine also acts as a pericyte relaxant drug, supporting its role in contrasting the pericyte contraction in response to myocardial ischemia [27,28]. It is worth mentioning that the 1976 T/C polymorphism of the adenosine 2A receptor gene may increase susceptibility to MVO, supporting the role of patients’ predisposition to the development of MVO [29].

Early studies of adenosine in the setting of acute myocardial infarction (AMI) in humans included the AMISTAD (Acute Myocardial Infarction Study of Adenosine), AMISTAD II, and ATTACC trials, which tested its intravenous administration [30,31,32]. The AMISTAD II trial was the first powered RCT to evaluate the clinical benefit of intravenous adenosine in patients with anterior STEMI. Patients with evolving anterior STEMI (6 h of onset of ischemic-type pain lasting at least 30 min, and with electrocardiographic evidence of anterior STEMI) receiving thrombolysis or primary PCI were randomized to a 3 h adenosine infusion (50 or 70 µg/kg/min) or placebo [31]. Although there were no differences in the primary endpoint (a composite of new or first re-hospitalization for congestive heart failure or death from any cause within six months), a dose–response relationship with final median infarct size was observed. The highest adenosine dose (70 µg/kg/min) significantly reduced infarct size, measured by technetium-99m sestamibi SPECT imaging between 120 and 216 h after randomization, with few adverse clinical events. The study led to different results compared to the first AMISTAD trial that showed a reduction in infarct size. A limitation of these studies was the use of an intravenous route for adenosine administration, which was responsible for a consistent proportion of patients experiencing marked systemic arterial hypotension, leading to use of lower adenosine doses. Additionally, the high variability in the duration of infusions other than the individualized dose adjustments limited the ability to attain to specific therapeutic protocols, thus affecting the generalizability of the results [33]. As a matter of fact, animal studies showed beneficial effects only with prolonged adenosine infusions, reinforcing the limitations in the design and conduct of these studies [34].

In this context, the attention was shifted to IC adenosine administration. The first trial tested high-dose IC adenosine in 51 patients with <70% ST-segment resolution after successful primary PCI with stent and treatment with abciximab [35]. Results were favorable, with higher ST-segment resolution and MBG and lower TFC and resistance indices. One year later, an RCT enrolling 448 STEMI patients (symptoms of chest pain suggestive of myocardial ischemia for at least 30 min, a time from onset of symptoms of <12 h before hospital admission, and an ECG showing ST-segment elevation of >0.1 mV in two or more leads) evaluated the effect of two bolus injections of IC adenosine (each of 120 μg) or placebo [36]. Results showed that IC adenosine after thrombus aspiration and after stenting of the infarct-related artery did not result in improved myocardial perfusion, based on computerized quantitative blush evaluation. Similar results were reported in other trials such as the REOPEN-AMI (Intracoronary Nitroprusside Versus Adenosine in Acute Myocardial Infarction) and the REFLO-STEMI (Reperfusion Facilitated by Local Adjunctive Therapy in STEMI) [37,38]. As for IV administration, optimal duration of drug infusion remained a point of debate. As pre-clinical studies showed no efficacy if adenosine was injected upstream of the occlusion site [33], a different site of adenosine administration (at the catheter site, at the site of PCI, or distal to the occlusion site) should be considered when interpreting these studies. Finally, results from meta-analyses are limited by the high heterogeneity of the studies included (different clinical presentation, different route of administration and duration and dose of infusion), without conclusive evidence [39,40].

### 4.2. Calcium Channel Blockers

Calcium channel blockers inhibit the influx of Ca^2+^ through calcium channels in myocardial cells, leading to coronary vasodilation and reduced myocardial oxygen demand [41]. Various CCBs, including verapamil, diltiazem, and nicardipine, have been explored for preventing or treating no-reflow [42,43]. Small-scale studies have produced mixed results, highlighting need for larger RCTs to draw definitive conclusions about their efficacy [44]. A meta-analysis of eight RCTs involving 494 patients demonstrated that IC injections of verapamil or diltiazem significantly reduced the incidence of no-reflow [45]. Nevertheless, the overall quality of the included studies was low due to the wide timeframe of inclusion (1997 to 2014), heterogeneity of the molecules investigated (diltiazem and verapamil) at different doses (ranging from 100 to 400 μg for verapamil to 0.5 mg), different durations of treatment (up to 6 months), and absence of high-sensitivity techniques to assess MVO. Moreover, the forest plot analysis revealed a low event rate in some studies, suggesting a potential bias due to small study effects.

### 4.3. Epinephrine

Epinephrine, acting as a β2-adrenergic receptor agonist, may promote coronary vasodilation at low doses, thus with the potential to prevent and treat MVO [46].

In the proof-of-concept RESTORE observational study, 30 consecutive STEMI patients with severe refractory coronary no-reflow (i.e., TIMI 0–1, MBG 0–1) were prospectively included to test the efficacy of IC epinephrine [47]. Compared to patients not receiving epinephrine, those undergoing IC epinephrine yielded better coronary flow patterns in terms of final TIMI flow. The COAR trial, which included 201 patients with acute coronary syndrome (ACS) of whom 95% had STEMI and angiographic evidence of no-reflow, showed a beneficial effect of IC epinephrine in enhancing TIMI flow and improving final CTFC, compared to adenosine [48]. Notably, this occurred without a significant impact on short-term clinical outcomes. Further observational studies have supported the evidence [49]. Again, (i) high variability in the dose used among studies, (ii) the design of the studies including different comparator groups (glycoprotein IIb/IIIa inhibitors, nitrates, adenosine) and mainly being non-randomized in their design, (iii) non-comprehensive evaluation of MVO confined to angiographic parameters, and (iv) the absence of studies powered to investigate the impact of treatment on clinical outcome have limited the evidence of epinephrine as an efficacious pharmacological approach to prevent and treat MVO.

### 4.4. Intracoronary Thrombolytic Agents

Several RCTs have evaluated the use of low-dose IC thrombolytic agents (urokinase, prourokinase, tenecteplase, and alteplase), suggesting their safety in the context of pPCI [50,51]. Nevertheless, these studies were limited by small sample size (half of them including less than 100 patients), high heterogeneity in inclusion and exclusion criteria, different doses and timing of infusion, concomitant use of thrombectomy, different strategies in background antiplatelet therapy, and, importantly, an absence of long-term clinical follow-ups. As a result, no evidence is currently available to support the routine use of IC thrombolytic therapy in STEMI patients.

### 4.5. Nicorandil

Nicorandil, a hybrid nitric oxide donor and ATP-sensitive potassium channel opener, exhibits vasodilatory and cardioprotective properties, potentially mimicking ischemic preconditioning [52]. The CHANGE trial, enrolling 238 high-risk STEMI patients experiencing episodes of chest pain persisting for at least 30 min (but no longer than 12 h) and ST-segment elevation of >0.1 mV in ≥2 electrocardiographic leads, found that a 6 mg bolus of nicorandil before pPCI, followed by continuous infusion at a rate of 6 mg/h, led to improved myocardial perfusion grade, increased LVEF, and reduced myocardial infarct size, as assessed by CMR imaging 5–7 days and 6 months after pPCI [53]. A recent meta-analysis of eight RCTs including 2055 STEMI patients also reported significant improvements in coronary blood flow and a reduced risk of MACE [54].

### 4.6. Oral Antiplatelet Agents

Among patients with ACS, potent oral P2Y_12_ inhibitors (prasugrel or ticagrelor) have shown direct cardioprotective effects in animal studies by reducing infarct size and MVO when administered before reperfusion [55]. Moreover, ticagrelor has been found to increase plasma adenosine levels, and small observational studies have suggested that high-dose clopidogrel pretreatment may also mitigate MVO [56]. Nevertheless, no studies have been conducted to specifically assess the effectiveness of these drugs on MVO evaluated trough high-sensitivity tools (CMR, PET, SPECT). The COMPARE CRUSH (Comparison of Pre-hospital Crushed Versus Uncrushed Prasugrel Tablets in Patients With STEMI Undergoing Primary Percutaneous Coronary Interventions) trial evaluated the impact of crushed versus integral prasugrel tablets on coronary reperfusion in 727 STEMI patients and symptom onset within 6 h [57]. The coprimary endpoints—TIMI 3 flow in the infarct-related artery before PPCI and complete ST-segment resolution 1 h after pPCI—showed no differences between groups. Shifting the focus to microcirculatory indices, the REDUCE-MVI (Reducing Micro Vascular Dysfunction in Acute Myocardial Infarction by Ticagrelor) randomized 110 patients with STEMI < 12 h after onset of symptoms, to receive a maintenance dose of ticagrelor or prasugrel after successful PCI [58]. Results showed no significant differences between ticagrelor and prasugrel in the primary outcome, intended as coronary microvascular injury at 1 month, determined with the index of microcirculatory resistance in the infarct-related artery.

### 4.7. Parenteral Antiplatelet Agents

Cangrelor, an intravenous P2Y_12_ inhibitor, achieves maximal platelet inhibition within minutes, offering timely platelet inhibition at the time of PCI [59]. Preclinical studies have shown that its administration at the time of reperfusion reduces infarct size by activating cytoprotective pathways (e.g., Akt, Erk 1/2) [60,61]. The phase 2 PITRI (Platelet Inhibition to Target Reperfusion Injury) trial tested whether cangrelor, initiated prior to the PPCI on top of oral ticagrelor, could have supported this hypothesis [62]. Results showed no significant differences in terms of infarct size (expressed as percentage of the left ventricle mass) by CMR, within the first week. Moreover, no differences in the extent of MVO were found. Acting on a different platelet target, glycoprotein IIb/IIIa inhibitors (GPIs) (abciximab, eptifibatide, tirofiban) block the final common pathway of platelet aggregation by inhibiting fibrinogen crosslinking, leading to rapid platelet inhibition, and have been found to reduce ischemic complications when used in the pre-procedural phase [63]. Overall, patients with STEMI who have received early abciximab administration before transfer to primary PCI rather than in the cathlab had more patent arteries before PCI and lower rates of death at 30-day follow-up [64,65]. Based on this assumption, they have been tested with the aim to assess their specific role in the prevention and treatment of MVO. In the AIDA (Abciximab Intracoronary versus Intravenously Drug Application in ST Elevation Myocardial Infarction) trial, IC administration of abciximab was not found to produce any advantage [63,66]. Conversely, the INFUSE-AMI (Intracoronary Abciximab and Aspiration Thrombectomy in Patients with Large Anterior Myocardial Infarction) trial, including 452 high-risk anterior STEMI patients (with symptom duration longer than 30 min and 1 mm or greater ST-segment elevation in two or more contiguous leads, new left bundle-branch block, and with anticipated symptom-onset-to-device time of 5 h or less) reported a significant reduction in infarct size (assessed by CMR) 30 days after the administration of an IC bolus of abciximab delivered to the infarct lesion by means of a perfusion balloon [67]. Finally, the recent REVERSE-FLOW trial investigated the use of GPIIb/IIa inhibitors as a strategy in case of CNR [68]. The study included patients with AMI and TIMI flow grade < 2 and found no differences in infarct size (primary endpoint), intended as % of LV mass assessed by CMR, confirming the absence of efficacy, as previously reported.

Given the variability in the current evidence toward negative results, other molecules are currently under investigation and include RUC-4 (zalunfiban), a subcutaneous GPIIb/IIIa antagonist [CELEBRATE trial (NCT04825743)], and selatogrel [SOS-AMI trial (NCT04957719)].

### 4.8. Sodium Nitroprusside

Sodium nitroprusside (SNP) is a potent vasodilator that releases nitric oxide (NO) and activates guanylate cyclase in vascular smooth muscle cells, increasing cyclic GMP levels [69]. Based on the assumption that it could counteract microvascular spasm and enhance microvascular perfusion through robust vasodilation of the coronary microcirculation, IC SNP, either alone or in combination with other agents (e.g., adenosine, tirofiban), has been evaluated in RCTs [70].

An early RCT explored the effect of IC SNP (60 μg) on post-PCI angiographic CTFC and ST-segment elevation resolution in 98 STEMI patients, revealing no significant improvement in coronary flow or myocardial tissue reperfusion [71]. Conversely, Zhao et al. evaluated IC SNP (100 µg) delivered via a thrombus aspiration catheter in 162 STEMI patients, demonstrating that SNP, in addition to tirofiban, significantly reduced post-procedural CTFC and increased the proportion of complete STR [72]. No impact on MACE was found. Within the same context, the REOPEN-AMI trial randomized 240 STEMI patients (symptom onset <12 h before enrollment, ST-segment elevation of at least 2 mm in two or more contiguous leads, and TIMI flow grade 0/1 at baseline angiography) to receive adenosine (120 mg as fast bolus followed by 2 mg given in 33 mL of saline over 2 min as slow bolus), SNP (60 mg as fast bolus followed by 100 mg given in 33 mL of 5% glucose over 2 min as slow bolus), or placebo [37]. Results showed that adenosine provided a higher ST-segment resolution >70%, compared to SNP and placebo. Similarly, the REFLO-STEMI (Reperfusion Facilitated by Local Adjunctive Therapy in ST-Elevation Myocardial Infarction) and RAIN-FLOW (Treatment of Slow-Flow After Primary Percutaneous Coronary Intervention With Flow-Mediated Hyperemia) trials found no differences between adenosine and SNP compared to placebo with regard to infarct size, MVO measured by CMR (REFLO-STEMI) and thermodilution-based minimal microcirculatory resistance [73,74]. When these studies were incorporated in meta-analyses, only angiographic benefits of SNP emerged, with no strong benefit on clinical outcomes or on additional metrics (SPECT, CMR, PET) of MVO assessment [75].

### 4.9. Other Pharmacological Strategies from Recent Randomized Trials

Despite extensive investigations based on different adjunctive therapies in STEMI, these recent randomized trials have not consistently demonstrated a clear benefit. In the COVERT-MI trial (Colchicine for Left Ventricular Infarct Size Treatment in Acute Myocardial Infarction), 192 patients with STEMI were randomized to evaluate whether colchicine could attenuate myocardial injury compared with placebo in those undergoing pPCI. Short-term, high-dose oral colchicine administered at the time of reperfusion did not reduce infarct size as assessed by CMR, nor did it significantly affect other indices of myocardial damage, including MVO or LV remodeling [76]. There was also no significant difference between the colchicine and placebo groups at 1 year regarding MACEs [77]. Among emerging strategies targeting reperfusion injury and microvascular dysfunction, the PULSE-MI randomized clinical trial tested a pre-hospital administration of a single 250 mg IV dose of methylprednisolone before pPCI in STEMI patients. This strategy did not significantly reduce final infarct size at three months (median 5% vs. 6% of LV mass; *p* = 0.24). Nevertheless, early CMR showed smaller acute infarct size, less MVO, and higher LVEF in the glucocorticoid group, without an increase in adverse events [78]. Finally, in the ASSAIL-MI randomized trial, early intravenous administration of the interleukin-6 receptor inhibitor tocilizumab during pPCI for STEMI significantly improved myocardial salvage and reduced the extent of MVO, while showing only a non-significant trend toward smaller infarct size. The therapy was well tolerated, with no increase in adverse events [79]. Overall, these contemporary randomized studies underscore the persistent challenge of translating promising cardioprotective concepts into durable reductions in infarct size and microvascular injury following pPCI in STEMI.

## 5. Interventional Prevention and Treatment of Microvascular Obstruction

The term “interventional treatment” refers to non-pharmacological therapeutic approaches that employ single or combined strategies to target the pathophysiological mechanisms of no-reflow and MVO. These strategies are implemented in the catheterization laboratory before, during, or immediately after PCI with the primary goal of preventing MVO. Table 2 shows tested interventional strategies to prevent and treat MVO.

### 5.1. Direct Stenting

Direct stenting is a procedural strategy which consists in implanting a stent without any pre-dilation and lesion preparation, avoiding plaque manipulation, which may contribute to distal embolization. The first RCT was conducted on 408 patients with AMI, showing no significant improvement in angiographic outcomes and no difference in hard clinical endpoints (death and recurrent MI) [80]. The DIRAMI trial showed no difference in angiographic outcomes and equipoise in 5-year clinical outcomes, along with an increase in in-stent restenosis [81]. With regard to CMR data that consistently allowed assessment of the rate of MVO with higher sensitivity, a subgroup analysis of the LIPSIA CONDITIONING trial, conducted on STEMI patients, widened the debate [80,82]. Patients were stratified according to the PCI technique performed (i.e., direct stenting vs. conventional stenting with pre-dilation) [83]. After matching for baseline and procedural characteristics, patients undergoing direct stenting had significantly lower MVO (assessed by CMR), with direct stenting being an independent predictor of reduced mortality. Nevertheless, it should be noted that it was a non-prespecified analysis, conducted in the context of an RCT that compared the efficacy and safety of a pre-conditioning strategy, and not directly focusing on a direct stenting strategy, at the operator’s discretion. A meta-analysis including 12 studies, of which 3 were RCTs [84], showed a beneficial effect of direct stenting on angiographical outcomes only among non-randomized studies, with the majority of them not reflective of the current clinical practice, without evidence on MVO, and without a clear trend toward improved hard clinical endpoints.

### 5.2. Specific Stent Platforms

Specific stent platforms have been developed to prevent distal embolization during pPCI, targeting one of the key mechanisms leading to MVO. Among these, the MGuard stent (InspireMD, Inc., Miami, FL, USA (6303 Waterford District Drive, Suite 215, Miami, FL 33126, USA)) features a bare-metal stent design covered with a polyethylene terephthalate micronet mesh to trap embolism-prone material [85]. In the MASTER trial, the MGuard stent demonstrated superiority over conventional bare-metal and drug-eluting stents in terms of complete ST-segment resolution (≥70%) and achieving post-procedural TIMI flow grade 3 among STEMI patients with symptoms lasting ≤12 h and ST-segment elevation >2 mm in ≥2 contiguous leads [86]. However, despite these promising angiographic results, patients treated with the MGuard stent experienced significantly higher rates of MACE at one year. This was driven by ischemia-related target lesion revascularization and poor long-term stent performance, undermining the feasibility of using this device to prevent distal embolization due to its counterbalanced safety issues [87]. Similarly, the STENTYS coronary stent (Stentys S.A., Paris, France) was developed as a self-expanding bare-metal stent with a nitinol platform and z-shaped design. Its self-apposing properties aimed to minimize barotrauma, reduce plaque disruption, and limit thrombus dislodgement, thereby potentially lowering the risk of CNR. Its clinical program was however discontinued following reports of high MACE at one-year follow-up, including increased stent thrombosis in the investigational group [88,89,90,91]. Moreover, no significant differences in TIMI flow grades or MBG were observed with the STENTYS stent, failing to support the primary hypothesis of the potential benefit in preventing and treating MVO.

### 5.3. Deferred Stenting

A two-step strategy involving initial reperfusion by balloon angioplasty and/or thrombus removal followed by stent implantation hours or days later has been proposed. This approach aims to reduce thrombus burden, allowing for adequate restoration of epicardial flow, but avoiding the adverse effects of immediate stenting [92]. The proof-of-concept DEFER-STEMI trial evaluated deferred stenting, performed 4 to 16 h after primary coronary angiography, and found significant benefits, including reduced incidences of no- or slow-reflow, fewer intraprocedural thrombotic events, improved TIMI flow grades at the end of the procedure, and a greater myocardial salvage index at six months [93]. Conversely, the DANAMI-3-DEFER trial, which randomized 1215 STEMI patients to standard PCI or deferred stenting, found no significant differences in clinical outcomes at two years [94]. Interestingly, a substudy indicated that deferred stenting resulted in lower incidences of distal embolization and no- or slow-reflow, with a particular benefit among high-risk subgroups (patients over 65 years of age, those presenting with an occluded culprit artery at admission, and those with a thrombus grade greater than 3) [95]. One possible explanation for these mixed findings is the timing of deferred stenting, which occurred at a mean of 48 h in the DANAMI-3-DEFER trial without specific angiographic criteria to guide the procedure. In contrast, the first group to report a minimalist immediate mechanical intervention using a simple guidewire or a small balloon to restore TIMI 2–3 flow in the infarct-related artery suggested postponing stenting for up to 7 days after achieving flow stabilization [96]. Whether this occurred in the DANAMI-3-DEFER trial remains unknown, and may have overshadowed the potential benefit of the deferred stenting strategy on hard clinical endpoints [97].

A 2018 meta-analysis of randomized controlled trials reported that deferred stenting improves angiographic outcomes by reducing no- or slow-reflow, although MVO rates were comparable. This highlights the variability and sensitivity of different metrics used to assess outcomes, including angiographic and non-angiographic measures [98]. Of note, the treatment effect was strongly correlated with a high thrombus burden at baseline angiography and the total stent length implanted in the culprit vessel, suggesting that patient selection and procedural optimization play crucial roles in the success of this strategy.

### 5.4. Aspiration Thrombectomy

A large body of studies have investigated the role of manual aspiration thrombectomy, reporting mixed angiographic and clinical outcome results. The TAPAS trial demonstrated improved angiographic outcomes with manual aspiration in STEMI patients (symptoms of acute myocardial ischemia lasting more than 30 min, onset < 12 h, and ST-segment > 0.1 mV in two or more leads on ECG), which translated into potential benefits in 30-day and 1-year outcomes [99]. However, the TOTAL and TASTE trials found no significant improvement in angiographic or clinical outcomes. Notably, in the TASTE trial, patients undergoing thrombectomy reported a doubled risk of ischemic stroke at 30 days in the aspiration group [99]. When data from these trials were pooled in an individual patient meta-analysis, the lack of beneficial effect from thrombectomy was reconfirmed [100]. It should be highlighted that manual thrombus aspiration is an operator-dependent technique. Therefore, while it can be a useful tool during pPCI, especially in cases of high thrombus burden [101], its routine use in STEMI patients is not supported [102].

### 5.5. Stent-Based Mechanical Thrombectomy Devices

Stent-based mechanical thrombectomy devices, also known as stent retriever systems, are self-expanding nitinol stent-based devices designed to restore blood flow immediately after deployment at the lesion level [103]. Capturing and removing atherothrombotic debris, they have the potential to minimize distal embolization. The first-in-man experience with the NeVa (Vesalio) mechanical thrombectomy device suggests safety and high rates of vessel recanalization and thrombus removal. The RETRIEVE-AMI (RETRIEVEr thrombectomy for thrombus burden reduction in patients with Acute Myocardial Infarction) trial enrolled 81 STEMI patients with TIMI thrombus grade > 4 and randomized them to PCI, manual aspiration-assisted PCI or stent-retrieve thrombectomy PCI. Both techniques significantly reduced present thrombus burden compared to no modification, without any differences in primary endpoint of pre-stent thrombus volume by optical coherence tomography between groups [104,105].

### 5.6. Distal Protection Devices

Distal protection devices are designed to capture debris while maintaining blood flow, aiming to prevent distal embolization of atherothrombotic material during PCI [8]. The EMERALD (Enhanced Myocardial Efficacy and Recovery by Aspiration of Liberated Debris) trial, which randomized 501 STEMI patients presenting within 6 h of symptom onset to PCI plus balloon occlusion and aspiration distal microcirculatory protection system or PCI alone, demonstrated that the distal protection system failed to reduce no-reflow and had no significant impact on final TIMI flow, final CTFC, MBG, ST-segment resolution > 70%, or infarct size [106]. Other RCTs have similarly shown that routine use of distal protection devices during primary PCI did not improve angiographic and clinical outcomes [107,108,109].

Importantly, technical challenges associated with their use, such as thrombus embolization during device placement, inability to have access to the target coronary branch, and risk of device entrapment, significantly limit their clinical utility.

### 5.7. Excimer Laser Coronary Atherectomy

Excimer laser coronary atherectomy uses IC catheters containing flexible optic fibers that release pulse waves to reduce thrombus burden through plaque debulking and thrombus vaporization, potentially minimizing the risk of distal embolization before stent implantation [110,111,112]. However its clinical efficacy in reducing MVO has not been rigorously tested in large-scale RCTs [113,114,115].

### 5.8. Pressure-Controlled Intermittent Coronary Sinus Occlusion (Picso)

PiCSO (Miracor Medical SA, Brussels, Belgium) is a percutaneous interventional procedure in which a balloon-tipped catheter is positioned in the coronary sinus and, through cyclical inflating and deflating of the balloon, transiently increases back-venous pressure in the coronary sinus, redistributing blood flow to the ischemic myocardium [116]. PiCSO represents a potential advancement in the treatment of MVO, aiming to enhance coronary perfusion and improve clinical outcomes [117]. Early investigations in patients with STEMI, such as the PREPARE-RAMSES and OxAMI-PICSO studies, indicated potential benefits [118,119]. Those benefits were accrued especially in patients with high pre-PCI IMR (above 40) values, with positive results both in anterior and inferior STEMI patients. However, the PICSO-AMI-I trial, which was expected to provide further insights on the topic, was prematurely discontinued by the sponsor [120]. It showed that PiCSO therapy did not reduce infarct size compared to conventional primary PCI in patients with anterior STEMI, and it was associated with increased procedural time and contrast medium use. These findings may be explained by suboptimal procedural conduct (i.e., half of the enrolled patients were maintained on PiCSO treatment for the optimal recommended time of 45 min; additionally, device position, and especially not-deep placement, could have resulted in device dislodgment), and also in patients’ selection, since the design allowed for the inclusion of patients with symptoms lasting up to 12 h before hospital arrival.

### 5.9. Supersaturated Oxygen

Intracoronary delivery of hyperoxic blood [i.e., Supersaturated Oxygen (SSO_2_)] has been associated with decreased endothelial edema, higher capillary vasodilatation, and decreased infarct size [121]. It consists in the delivery of 60 min of hyperoxemic blood into the infarct zone territory following successful reperfusion. The efficacy in reducing infarct size has been attributed to the ability of SSO_2_ to achieve significantly higher levels of dissolved oxygen in plasma available for diffusion into the ischemic zone as well as a counter-intuitive reduction in harmful free radical generation. The first RCT, the AMIHOT (Acute Myocardial Infarction with Hyperoxemic Therapy) trial, found a reduction in infarct size, without differences in MACE compared to normoxemic blood reperfusion [122]. A pool of patients from AMIHOT and AMIHOT II, a second RCT including patients with anterior AMI within six hours from symptom presentation, received SSO_2_ infusion into the infarct-related artery following successful primary PCI, showing a significant reduction in infarct size [123]. Higher rates of hemorrhagic events with SSO_2_ and a trend toward higher rates of stent thrombosis were reported. To overcome the limitation of selective infusion catheters, the IC-HOT trial tested the application of SSO_2_ at the left-main level, showing the feasibility and safety of the approach [124]. The results of this study led to U.S. Food and Drug Administration approval of SSO_2_ therapy for patients with anterior STEMI undergoing pPCI within 6 h of symptom onset. Recently, a pooled analysis of individual patient data from nine studies, comparing the outcomes of 894 patients with anterior STEMI who underwent LAD PCI with and without treatment with SSO_2_ (90 vs. 784), showed that SSO_2_ therapy was independently associated with a lower extent of MVO (assessed through CMR within 10 days after pPCI) compared with no SSO_2_ therapy [125]. Similarly, pre- and post-procedural angiography-derived indices of microcirculatory resistance (angio-IMR) were evaluated in 46 patients with anterior STEMI who underwent successful pPCI of the left anterior descending artery followed by SSO_2_ therapy [124,126]. Results showed that angio-IMR was significantly reduced after SSO_2_ in patients with higher pre-SSO_2_ angio-IMR values (>40), and correlated with CMR-derived infarct size and MVO extent at day 4 and at day 30 [127]. Taken together, these findings suggest that SSO_2_ may be a promising option for the prevention and treatment of MVO.

### 5.10. Other Treatment Strategies

Remote ischemic conditioning (RIC) and postconditioning (PostC) have emerged as promising strategies to mitigate ischemia–reperfusion injury, a key mechanism in the development of MVO. RIC involves cycles of brief ischemia and reperfusion applied to a tissue or organ remote from the heart, either before (pre-conditioning) or during (per-conditioning) ischemia. PostC, on the other hand, is realized through short, repeated episodes of iatrogenic myocardial ischemia followed by reperfusion, immediately after pPCI. Both approaches have demonstrated efficacy in reducing infarct size in experimental models, but clinical evidence remains inconsistent [128]. The first randomized evaluation of PostC, conducted more than a decade ago, showed positive results. In a study of 50 patients undergoing pPCI for a first STEMI with TIMI flow grade 0–1, PostC significantly reduced infarct size as assessed by CMR within 96 h of reperfusion [129]. Similarly, the LIPSIA CONDITIONING trial demonstrated that combining intrahospital RIC and PostC with pPCI significantly improved myocardial salvage compared to controls or PostC alone [130]. Furthermore, the combination of RIC and PostC was associated with a reduction in major adverse cardiovascular events (MACE) over a median follow-up of 3.6 years, primarily due to a lower incidence of new-onset heart failure [131]. Despite these promising findings, subsequent studies have reported conflicting results. The DANAMI-3-iPOST trial and a substudy of the CONDI-2/ERIC-PPCI trial found no improvement in clinical outcomes with routine application of PostC or RIC [132,133]. Similarly, a National Heart, Lung, and Blood Institute-sponsored trial evaluated the early and long-term effects of PostC in 122 STEMI patients with ischemic times between 1 and 6 h, and reported no early benefits in terms of infarct size, myocardial salvage index, or left ventricular function [134].

Other strategies tested to reduce MVO have yielded limited or conflicting evidence. These include statins, beta-blockers, cyclosporine A, and carperitide. Similarly, hypothermia, hypothesized to reduce metabolic demand and inflammatory response, has not reported favorable results [8,10]. In the COOL AMI EU pilot trial, rapid systemic endovascular cooling with the ZOLL Proteus system achieved deep pre-reperfusion hypothermia (33.6 °C) with a modest pPCI delay. Infarct size was numerically smaller in the “cooled” group but not statistically significant. The procedure proved feasible and generally safe, with only transient atrial fibrillation (32% vs. 8%) and two stent thromboses likely related to procedural or pharmacologic factors [135]. In contrast, the EURO-ICE trial evaluated selective IC hypothermia (30–33 °C for 7–10 min before and 10 min after reperfusion) in 200 patients with anterior STEMI. The technique demonstrated a good safety profile but failed to reduce infarct size or microvascular injury. At three months, MVO was 2.81 ± 4.46% versus 2.25 ± 5.34% of LV mass (*p* = 0.44), with comparable rates of intramyocardial hemorrhage between groups [136]. Further investigations are needed to optimize timing, target temperature, and duration of hypothermia to achieve effective microvascular protection and infarct size reduction.

## 6. Ongoing Trials and Future Directions

### 6.1. New Trials Exploring Novel Cardioprotective Approaches in STEMI

Across the STEMI landscape, a new generation of clinical trials is reshaping reperfusion therapy by testing adjunctive device-based and pharmacologic strategies aimed at further reducing infarct size and MVO beyond the benefit achieved with pPCI alone. Analyzing the new device-based strategies, the STEMI-DTU program (NCT03947619) investigates the safety and effectiveness of LV unloading with the IMPELLA^®^ CP System prior to reperfusion, versus current standard of care, in reducing infarct size and heart failure-related clinical events [137]. On the other hand, other trials are re-evaluating the RIC concept; the RIC-AFRICA trial (NCT04813159) is a multicenter, sham-controlled randomized study enrolling STEMI patients mainly treated with thrombolytic therapy within 24 h of symptom onset across seven African countries. It investigates whether RIC, induced by brief cycles of upper-arm ischemia and reperfusion initiated before thrombolysis and continued for two days, can reduce 30-day mortality and early heart failure [138]. Another trial is testing RIC: the RIP-HIGH trial (NCT04844931), which is a randomized, two-arm study evaluating the combined effect of RIC and PostC compared with standard care in STEMI patients undergoing pPCI. This trial focuses on patients with significant hemodynamic compromise (Killip class ≥ 2), in whom mortality and infarct size remain substantial despite timely reperfusion. By applying short cycles of transient arm ischemia and brief interruptions of coronary flow after reperfusion, the study seeks to attenuate ischemia–reperfusion injury and limit myocardial damage [139]. Regarding SSO2 therapy, the HOT-AAMI trial (NCT06742684) investigates IC SSO_2_ therapy administered immediately after PCI in anterior STEMI. This randomized multicenter study focuses on clinical outcomes such as mortality and heart-failure progression, building on earlier studies that suggested potential benefits of SSO_2_ in improving myocardial recovery [140]. Finally, attention has also turned to ultrasound-based reperfusion techniques such as the REDUCE trial (ACTRN12620000807954), which is a multicenter, sham-controlled study designed to assess the efficacy of sono-thrombolysis delivered both before and after pPCI in STEMI. Using diagnostic ultrasound combined with microbubble contrast, this approach aims to enhance microcirculatory flow and reduce infarct size. The primary endpoint is infarct size measured by CMR, with secondary outcomes including LV function and six-month clinical follow-up [141].

On the pharmacologic front, the UPFRONT-STEMI trial (NCT05393557) targets microvascular dysfunction through an early “microcirculatory preparation” strategy combining GP IIb/IIIa inhibitors, nitroglycerin, and verapamil administered before full reperfusion. This is followed by staged restoration of flow using repeated balloon inflation and deflation, aiming to prevent distal embolization and reduce both angiographic no-reflow and MVO [142]. Other studies are focusing on the reduction of oxidative stress and the modulation of inflammatory responses in STEMI patients. The IOCYTE AMI-3 trial (NCT04837001) is evaluating intravenous FDY-5301 in patients with anterior STEMI undergoing pPCI, in a large phase 3, double-blind, placebo-controlled multicenter design. FDY-5301 is a sodium-iodide-based agent that could limit ischemia–reperfusion injury by dampening the inflammatory response that follows reperfusion [143]. The COOPERATION trial (NCT04912518) is a double-blind, multicenter, randomized, placebo-controlled study investigating the cardioprotective potential of dexmedetomidine in patients with anterior STEMI undergoing pPCI within six hours of symptom onset. Intravenous dexmedetomidine is administered from the start of PCI and stopped at procedure completion, with saline infusion used as placebo. The primary endpoint is infarct size measured by CMR [144]. Finally, the NORMAL trial (NCT06787430) is a prospective, double-blind, randomized study evaluating whether IC nicorandil improves microvascular function in STEMI patients undergoing primary PCI. Patients are randomized 1:1 to nicorandil or placebo, with two boluses administered after initial flow restoration and before stent implantation. Microvascular function is assessed using the angiography-derived index of microcirculatory resistance (AMR) [145].

Collectively, these ongoing investigations highlight an expanding therapeutic frontier encompassing device-based and pharmacological strategies, all converging toward the same translational goal of reducing MVO and maximizing myocardial salvage in patients with STEMI.

### 6.2. Future Directions

Looking ahead, a precision-medicine approach is essential. Standardizing diagnostic methods, tailoring therapies to the underlying mechanisms of MVO, and stratifying patients based on risk profiles and individual susceptibilities are critical steps to optimize outcomes. Future RCTs should prioritize comprehensive evaluations of therapeutic efficacy, encompassing not only surrogate endpoints but also hard clinical outcomes, to build a stronger evidence base.

By addressing these limitations and taking advantages of the advancements in diagnostic and therapeutic modalities, the field can progress toward more effective strategies for the prevention and treatment of MVO, ultimately improving outcomes for STEMI patients.

## 7. Conclusions

Various pharmacological and interventional strategies have been investigated to mitigate MVO in STEMI patients undergoing pPCI. While several promising findings have emerged, these have not been translated into improved clinical outcomes in large RCTs. This discrepancy may be attributed to the multifactorial nature of MVO pathophysiology, variability in therapeutic responses influenced by drug dosages, and the effects of background antithrombotic therapies. Of note, many studies were conducted over a decade ago, often including patients who had already received thrombolytics or who experienced wide time delays to pPCI. Additionally, most trials were not powered to assess hard clinical endpoints.

Lack of standardization in diagnostic methods has further contributed to inconsistencies in the reported incidence of MVO. While angiographic indices are widely used due to their accessibility, they offer limited sensitivity, especially given the dynamic nature of MVO, which can evolve hours or days after pPCI. Advanced diagnostic tools, such as CMR, provide greater sensitivity and valuable prognostic information, yet remain underutilized in both clinical practice and RCTs.

Despite these challenges, notable advances have been made. Intracoronary therapies and emerging approaches, such as SSO_2_, have shown encouraging results in specific patient subgroups or in improving surrogate outcomes. However, these findings require validation through large-scale, methodologically robust trials that address current evidence gaps.

## Figures and Tables

**Table 1:** Current pharmacological strategies tested for the treatment of microvascular obstruction.

Drug	Route of Administration	Timing of Administration	Mechanism
**Calcium Channel Blockers ** (adenosine, diltiazem, verapamil)	Intravenous or Intracoronary *	During Primary PCI	Vasoconstriction is considered a key contributor and is mediated by several receptors in epicardial conduit arteries and microvessels (such as α1-adrenergic and α2-adrenergic receptors) and by several signaling molecules (such as endothelin, serotonin and thromboxane.
**Sodium Nitroprusside**	Intracoronary	During Primary PCI	
**Nicorandil**	Intravenous	Pre-PCI, During Primary PCI, Post-PCI	
**Epinephrine**	Intracoronary	During Primary PCI	
**Glycoprotein IIb/IIIa Inhibitors** (abciximab, eptifibatide, tirofiban)	Intracoronary or Intravenous	During Primary PCI, Post-PCI **	Reduce thrombus burden by promoting its dissolution and avoiding new thrombus formation at the site of the lesion. Antithrombotic agents can prevent and reduce distal embolization of thrombotic material.
**Intravenous P2Y_12_ receptor inhibitor**	Intravenous	During Primary PCI	
**Intracoronary thrombolytic agents** (urokinase, prourokinase, tenecteplase, and alteplase)	Intracoronary	During Primary PCI	
**Oral P2Y_12_ Receptor Inhibitors** (prasugrel, ticagrelor)	Oral	Pre-PCI	

* Adenosine; ** post-PCI administration is made by intravenous route. Abbreviations: PCI = percutaneous coronary intervention.

**Table 2:** Current interventional strategies tested for the treatment of microvascular obstruction.

Mechanical Devices		Characteristics
**Specific Stent Platforms**	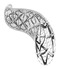	Balloon-expandable bare-metal stent with polyethylene terephthalate (PET) micronet mesh or self-expanding stents
**Thrombectomy**	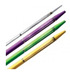	Catheters connected to syringe to aspirate thrombotic component from the culprit lesion using vacuum force.
**Stent-Based Mechanical Thrombectomy Devices**	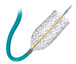	Self-expanding nitinol stent-based devices designed to restore blood flow immediately after deployment at the lesion level. Capturing and removing atherothrombotic debris, they have the potential to minimizing distal embolization.
**Distal Protection Devices**	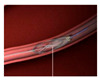	Filter with micro-holes to capture atherothrombotic debris distally to the culprit lesion while allowing blood flow maintenance.
**Excimer laser coronary atherectomy**	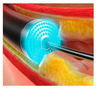	Catheters that release pulse waves by means of flexible optic fibers that reduce thrombus burden by plaque debulking and thrombus vaporization.
**Pressure-controlled intermittent coronary sinus occlusion (PiCSO)**	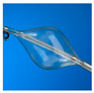	Balloon-tipped catheter that increase back-venous pressure in the coronary sinus, redistributing blood flow from the remote to the ischemic myocardium.
**Supersaturated Oxygen**	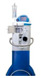	Intracoronary delivery of hyper-oxygenated blood.

## Data Availability

No new data were created or analyzed in this study. Data sharing is not applicable to this article.

## Abbreviations

AMI = Acute Myocardial Infarction; CMR = Cardiac Magnetic Resonance; ECG = Electrocardiogram; IC = Intracoronary; MACE = Major Adverse Cardiac Events; MCE = Myocardial Contrast Echocardiography; MVO = Microvascular Obstruction; PCI = Percutaneous Coronary Intervention; PET = Positron Emission Tomography; PiCSO = Pressure-Controlled Intermittent Coronary Sinus Occlusion; RCTs = Randomized Controlled Trials; SNP = Sodium Nitroprusside; SPECT = Single-Photon Emission Computed Tomography; SSO_2_ = Supersaturated Oxygen; STEMI = ST-Elevation Myocardial Infarction; TIMI = Thrombolysis in Myocardial Infarction.
